# Exploring Social Inequalities in Post-pandemic Labour Market Shifts and Job Dissatisfaction in Barcelona: Insights From a Southern European City

**DOI:** 10.7759/cureus.80847

**Published:** 2025-03-19

**Authors:** Carles Pericas, Carles Vilaplana-Carnerero, Héctor Martínez-Riveros, Lucia Artazcoz, Xavier Bartoll-Roca, Dolores Álamo-Junquera, Marta M Arcas, Cristina Rius, Maria Grau

**Affiliations:** 1 Department of Medicine, School of Medicine and Health Sciences, University of Barcelona, Barcelona, ESP; 2 Epidemiology Service, Public Health Agency of Barcelona (ASPB), Barcelona, ESP; 3 Epidemiology and Public Health, Biomedical Research Networking Centre (CIBER), Madrid, ESP; 4 Sant Pau Research Institute (IR Sant Pau), Hospital de la Santa Creu i Sant Pau, Barcelona, ESP; 5 Service for the Promotion of Quality and Bioethics, General Directorate of Health Planning and Regulation, Department of Health, Government of Catalonia, Barcelona, ESP; 6 Centre of Epidemiological Studies on Sexually Transmitted Infections and AIDS of Catalunya (CEEISCAT) Department of Health, Government of Catalonia, Badalona, ESP; 7 Germans Trias i Pujol Research Institute (IGTP), Campus Can Ruti, Badalona, ESP; 8 Department of the Public Health Observatory, Public Health Agency of Barcelona (ASPB), Barcelona, ESP; 9 Department of Experimental and Health Sciences, Faculty of Health and Life Sciences, Universitat Pompeu Fabra (UPF), Barcelona, ESP; 10 Health Information Systems and Evaluation Service, Public Health Agency of Barcelona (ASPB), Barcelona, ESP; 11 Catalan Institute of Health (ICS), Government of Catalonia, Sabadell, ESP; 12 Quality and Research Service, Mollet Healthcare Foundation, Hospital de Mollet, Mollet del Vallès, ESP

**Keywords:** covid-19 pandemics, job dissatisfaction, occupational health, public-health, social inequalities

## Abstract

The COVID-19 pandemic exacerbated labour market inequalities, disproportionately affecting low-paying, precarious jobs and vulnerable groups, while also accelerating shifts such as telecommuting, which had mixed impacts on job satisfaction. This study aimed to assess social inequalities in changes in the labour market as well as job dissatisfaction among workers in the city of Barcelona between 2016 and 2022.

A comparison of two population-based cross-sectional studies was carried out, using data from the Barcelona Health Surveys (BHS) of 2016-17 and 2021-22. Data sampling was weighted to ensure representativeness. A descriptive analysis was carried out for sociodemographic and work-related variables and multivariate weighted linear regressions were adjusted by potential confounders between year of the sample and job dissatisfaction.

Job status improved and job dissatisfaction decreased in both sexes between 2016 and 2022. Having a non-manual job entailed a decrease in job dissatisfaction for women and men (-0.09; 95% CI -0.16 to -0.03 and -0.10; -0.16 to -0.04 respectively). Among women born outside Spain, job dissatisfaction increased (0.09; 0.02 to 0.16). Job dissatisfaction decreased among men with a full-time contract (-0.11; -0.21 to -0.02). In both sexes, having no contract was associated with an increase in job dissatisfaction.

There were improvements in the labour market of Barcelona. Job dissatisfaction decreased, but not among migrant women or individuals with manual jobs and precarious contractual situations. Policies developed in recent years might have mitigated the negative effects of COVID-19 on the labour market.

## Introduction

Social determinants of health (SDH) are defined as the conditions in which individuals are born, grow, live, work, and age. Work is a widely recognized SDH, having a significant impact on individual and population health [1-3].

The characteristics of the job being performed, such as the skills required, the type of contract or whether a person works part-time or full-time, also determine the health status and well-being of individuals, even in the absence of direct illness or injury [4,5]. The balance between paid and domestic labour can also entail adverse consequences on the well-being of those with a major burden of domestic work, most of them being women [6,7]. Frameworks such as the demand-control-support model and the effort-reward imbalance model exemplify how working conditions influence health. High job demands paired with low control or inadequate support can lead to less satisfaction at work and stress-related health outcomes. Moreover, an imbalance between the effort put into work and the rewards received, whether it’s salary, recognition, or opportunities, can lead to dissatisfaction and decreased quality of life [8,9]. Conversely, better job satisfaction has been linked to improved mental health and overall well-being. This dichotomy highlights the importance of analysing job dissatisfaction as a key determinant of health, particularly in the context of shifting labour markets [10].

The COVID-19 pandemic brought unprecedented disruption to the labour market, with job losses concentrated among those in precarious or unstable work situations, particularly during 2020 [11,12]. As the pandemic progressed, it also became more evident that certain workers were disproportionately affected by COVID-19, specifically those with low-paying jobs [2]. These jobs with more risk of exposure to SARS-CoV-2 had a higher proportion of ethnic minorities, migrants, or individuals with low socioeconomic status, highlighting occupational segregation that placed already vulnerable groups in an even more precarious situation [13,14]. In Spain, during the first waves of the pandemic, essential workers exhibited higher rates of infection, while healthcare personnel reported a significant mental health burden during the pandemic [15,16].

In addition to the loss of paid jobs and the direct exposure to SARS-CoV-2, the pandemic also entailed significant shifts in the nature of work, notably through the rapid implementation of telecommuting to adhere to containment measures [17]. While telecommuting has been linked to improved job satisfaction in some cases, it often impaired work-life balance [2,18]. This is particularly true for women and non-skilled workers, who were less likely to experience the benefits of working from home [2,19].

Legislative measures in Spain sought to address the aforementioned challenges, and broader labour market reforms, including minimum wage increases and the 2021 labour reform, introduced structural changes with potential implications for worker satisfaction and inequality. Specifically, the Ley 10/2021, de 9 de julio, de trabajo a distancia (Remote Work Act, 2021) regulated remote work, while the Real Decreto 152/2022, de 22 de febrero (Royal Decree 152/2022, 2022) established the minimum wage for 2022. Additionally, the Real Decreto-Ley 32/2021, de 28 de diciembre (Royal Decree-Law 32/2021, 2021) introduced urgent measures for labour reform, employment stability, and labour market transformation [20-22].

Barcelona, as a major metropolitan area with a diverse labour market, likely experienced similar post-pandemic shifts to those described. The city’s economy relies heavily on the service sector, tourism, and cultural industries, all of which were significantly impacted by the pandemic [23]. However, evidence on how these changes influenced job satisfaction and dissatisfaction among its residents remains limited.

This study aims to assess the post-pandemic changes in the labour market as well as job dissatisfaction in a population-based sample of individuals in the city of Barcelona between 2016 and 2022, stratifying by sex and analysing how sociodemographic characteristics could be related to such changes.

## Materials and methods

Study design and population

This study is a comparison of two population-based cross-sectional studies, using data from the Barcelona Health Survey (BHS). The BHS is a validated tool conducted every five years to compile information about the health and health-related behaviours of the population, as well as aspects related to SDH in Barcelona. For this study, both the 2016-2017 and 2021-2022 BHS were used, each with a representative sample of 4000 non-institutionalized citizens aged 15 years or older. All participants were surveyed through computer-assisted face-to-face interviews conducted by trained interviewers in the respondents’ homes. The BHS sample is selected from the municipal census through a simple randomization process with sex and age quotas to properly represent the population structure of each district. All results are then weighted through post stratification weights to restore the representativeness of the total sample [24].

For this study, only the working population aged 16-64 was included. Children below 16, students, unemployed and retired individuals were excluded from the sample.

Ethical considerations

The 2016-2017 and 2021-2022 BHS are official surveys within the 2016 and 2021 Statistical Action Plan of Catalonia, respectively, and comply with the Spanish 15/1999 and European 2016/679 data protection laws. Surveyed individuals were asked for their explicit informed consent to participate [24]. This study was reviewed and approved by the Bioethics Commission of the University of Barcelona (IRB00003099).

Variables and measurement instruments

Sociodemographic Variables

Sociodemographic characteristics included sex assigned at birth (woman or man), age, place of birth (in Spain or outside Spain), education level (university studies or not), social class (non-manual or manual job) and lack of financial strain.

Lack of financial strain was determined based on the participants’ answer to the question “Based on your household’s net monthly income, how do you normally make ends meet?”. Responses were graded on a scale from 1 to 6, with 1 indicating “with a lot of difficulty” and 6 “with no difficulty at all”. This variable was treated as continuous.

Work-Related Variables

Employment conditions were assessed through job status, which encompassed the following categories: long-life contract, temporary contract, no contract, self-employment, and company owner. The variable “Full-time contract” distinguished between individuals with full-time contracts and those without.

Working conditions were assessed using the variable “Hours of paid work per week”, treated as continuous, and the variable “Hours of domestic labour per week”, categorised into discrete ranges: 0 hours, 1 to 7 hours, 8 to 14 hours, 15 to 21 hours, and 22 hours or more. Job dissatisfaction was determined based on the participants’ answer to the question: “Generally speaking, are you satisfied with your job conditions?”. Responses were graded on a scale from 1 to 4, with 1 indicating “very satisfied” and 4 “very dissatisfied”. This variable was treated as continuous.

Data analysis

First, a descriptive analysis was carried out with continuous variables presented as means and standard deviations and categorical variables as frequencies and proportions. To compare both surveys, weighted Student t-tests and weighted chi-square tests were used to compare means and proportions, respectively. 

A multivariate weighted linear regression was adjusted for potential confounders that presented a significant association with the independent variable (year of the sample) and with the dependent variable (job dissatisfaction). The effect modification of the relationship between the year of the sample and job dissatisfaction for different variables was tested using the -2 log-likelihood test of nested models with and without interaction terms. Multivariate weighted linear regressions stratified by the variables that showed significant interactions were performed for all variables that presented a significant interaction.

All statistical analyses were performed with the R Statistical Package (R Foundation for Statistical Computing, Vienna, Austria; V.4.2.2).

## Results

The total sample comprised 3,713 individuals (1,780 from the 2016-2017 BHS and 1,933 from the 2021-2022 BHS), of whom 49.8% (n=1,853) were women. Table 1 presents the comparison of means and proportions for continuous and categorical variables, stratified by sex and survey year.

**Table 1:** Descriptive analysis of the sample, stratified by sex and year of the survey (N=3723) *Statistical significance: p-value < 0.05. Chi2 test for categorical variables and T-student test for continuous variables.

	Women (N=1853)			Men (N=1860)		
	2016 N=887	2021 N=966	p-value*	2016 N=893	2021 N=967	p-value*
Age, mean (SD)	42 (11)	42 (12)	0.700	42 (11)	42 (12)	0.470
Social class (non manual), n (%)	355 (38.8)	445 (45.5)	0.004	322 (35.1)	448 (46.0)	<0.001
Born outside Spain, n (%)	283 (31.1)	313 (32.1)	0.625	251 (27.4)	326 (33.6)	0.003
Lack of financial strain, mean (SD)	3.79 (1.10)	3.86 (1.20)	0.171	3.74 (1.07)	3.93 (1.17)	0.013
Job status, n (%)						
Lifelong contract	574 (64.9)	666 (68.9)	0.115	523 (58.8)	632 (66.5)	0.006
Temporary contract	153 (18.1)	136 (14.9)		131 (15.4)	116 (12.5)	
No contract	33 (3.1)	38 (4.0)		22 (2.4)	21 (2.2)	
Self-employed	89 (9.3)	92 (9.1)		156 (16.8)	127 (12.1)	
Company owner	27 (3.2)	22 (2.1)		50 (5.2)	60 (5.5)	
Full-time contract, n (%)	653 (73.6)	711 (72.7)	0.709	784 (87.4)	840 (85.9)	0.399
Paid labour hours per week, mean (SD)	36.0 (11.2)	35.8 (11.4)	0.711	40.7 (11.1)	39.5 (10.3)	0.021
Hours of domestic labour per week, n (%)						
0 hours	64 (7.2)	16 (1.5)	<0.001	159 (17.1)	41 (4.2)	<0.001
1 to 7 hours	206 (22.5)	241 (24.6)		396 (44.8)	418 (43.4)	
8 to 14 hours	338 (38.8)	379 (39.5)		267 (30.2)	336 (35.2)	
15 to 21 hours	171 (19.3)	239 (24.5)		52 (6.1)	125 (12.8)	
>= 22 hours	100 (11.2)	80 (9.0)		14 (1.4)	20 (2.0)	
Job dissatisfaction, mean (SD)	2.05 (0.65)	1.95 (0.68)	0.031	1.99 (0.64)	1.91 (0.67)	0.004

Sociodemographic analysis revealed that women in non-manual jobs were more prevalent in the 2021-2022 BHS (38.8% vs. 45.5%; p=0.004). Among men surveyed in 2021-2022, a higher proportion worked in non-manual jobs (35.1% vs. 46.0%; p<0.001), were born outside Spain (27.4% vs. 33.6%; p=0.003), and reported lower financial strain (p=0.013) compared to the 2016-2017 BHS.

Employment conditions also differed significantly among men. A higher proportion in the 2021-2022 BHS held long-life contracts (66.5% vs. 58.8%) or owned a business (5.5% vs. 5.2%), while fewer had temporary contracts (15.4% vs. 12.5%) or were self-employed (16.8% vs. 12.1%) compared to the 2016-2017 BHS.

Additionally, there was a significant decrease in the average number of paid hours that men worked each week (40.7 vs 39.5; p=0.021). There were also significant changes in the distribution of hours that both women and men dedicated to domestic labour. Most notably, the proportion of men who dedicated 0 hours to domestic labour decreased from 17.1% to 4.2%.

Table 2 presents the composition of dissatisfaction groups across sociodemographic and work-related variables, stratified by sex. Among women who reported being dissatisfied or very dissatisfied with their job conditions, a higher proportion were born outside Spain (40.9% and 34.5%, respectively; p<0.001). In contrast, women who reported being very satisfied or satisfied with their job conditions had a higher proportion of non-manual workers (48.8% and 42.5%, respectively; p=0.002). A similar pattern was observed among men, with a higher proportion of non-manual workers in the very satisfied and satisfied groups (49.9% and 39.5%, respectively; p<0.001).

**Table 2:** Job dissatisfaction level by sociodemographic and work-related variables stratified by sex *Statistical significance: p-value < 0.05. Chi2 test for categorical variables and T-student test for continuous variables.

		Women (N=1831)				
		1- Very satisfied	2- Satisfied	3- Unsatisfied	4- Very unsatisfied	p-value*
		N=368	N=1163	N=249	N=51	
Age, mean (SD)		42 (12)	43 (12)	42 (12)	40 (11)	0.554
Social class (non-manual), n (%)		188 (48.6)	498 (42.5)	94 (35.8)	13 (27.2)	0.002
Born outside Spain, n (%)		99 (25.5)	371 (31.3)	99 (40.9)	19 (34.5)	<0.001
Lack of financial strain, mean (SD)		4.10 (1.05)	3.88 (1.11)	3.30 (1.27)	3.14 (1.17)	<0.001
Job status, n (%)						
Long-life contract		250 (68.9)	806 (68.8)	142 (57.8)	30 (61.2)	<0.001
Temporary contract		45 (12.8)	183 (16.5)	52 (22.0)	8 (15.8)	
No contract		7 (2.0)	40 (3.2)	19 (6.6)	5 (8.2)	
Self-employed		41 (10.1)	97 (8.1)	31 (11.3)	7 (12.2)	
Company owner		22 (5.3)	24 (2.3)	3 (1.4)	0 (0.0)	
Full-time contract, n (%)		281 (74.8)	875 (75.0)	160 (63.4)	35 (71.1)	0.009
Paid labour hours per week, mean (SD)		35.8 (10.8)	36.1 (10.6)	34.6 (14.3)	37.4 (14.6)	0.627
Hours of domestic labour per week, n (%)						
0 hours		14 (3.6)	47 (3.9)	14 (5.7)	5 (9.8)	0.226
1 to 7 hours		87 (22.7)	289 (24.2)	52 (20.5)	15 (33.2)	
8 to 14 hours		149 (41.9)	450 (38.5)	95 (40.2)	19 (38.8)	
15 to 21 hours		79 (21.0)	269 (23.3)	54 (21.0)	7 (12.1)	
>= 22 hours		37 (9.9)	102 (9.7)	34 (12.5)	5 (6.2)	
		Men (N=1842)				
		1- Very satisfied	2- Satisfied	3- Unsatisfied	4- Very unsatisfied	p-value*
		N=406	N=1184	N=208	N=44	
Age, mean (SD)		41 (13)	43 (11)	41 (12)	42 (11)	0.920
Social class (non-manual), n (%)		209 (49.9)	471 (39.5)	73 (35.6)	11 (26.0)	<0.001
Born outside Spain, n (%)		112 (27.0)	374 (31.6)	70 (32.6)	15 (31.1)	0.341
Lack of financial strain, mean (SD)		4.36 (1.04)	4.00 (1.06)	3.53 (1.22)	3.09 (1.63)	<0.001
Job status, n (%)						
Lifelong contract		237 (60.0)	776 (65.8)	114 (57.1)	20 (46.6)	<0.001
Temporary contract		48 (12.5)	144 (12.8)	47 (22.5)	8 (18.7)	
No contract		8 (2.1)	18 (1.5)	10 (4.9)	7 (14.7)	
Self-employed		63 (14.2)	180 (14.7)	27 (11.2)	8 (17.6)	
Company owner		42 (9.1)	58 (4.5)	8 (3.3)	1 (2.4)	
Full-time contract, n (%)		358 (87.7)	1066 (89.5)	156 (73.6)	36 (83.6)	<0.001
Paid labour hours per week, mean (SD)		40.0 (11.8)	40.3 (9.6)	38.6 (12.7)	40.5 (16.2)	0.396
Hours of domestic labour per week, n (%)						
0 hours		44 (10.7)	129 (10.3)	21 (10.1)	6 (13.7)	0.169
1 to 7 hours		163 (39.7)	537 (45.6)	87 (44.2)	22 (50.9)	
8 to 14 hours		134 (33.1)	380 (32.6)	74 (35.2)	9 (20.5)	
15 to 21 hours		51 (12.9)	105 (9.0)	15 (6.6)	4 (8.7)	
>= 22 hours		9 (2.4)	18 (1.4)	5 (1.9)	2 (4.7)	

For both women and men, a significant negative gradient was observed between lack of financial strain and job dissatisfaction. Individuals with lower financial strain were less likely to report dissatisfaction.

Significant differences in job dissatisfaction were observed across the entire sample concerning the variables "job status" and "full-time contract". Both women and men who were very satisfied or satisfied with their job conditions had a higher proportion of individuals with long-life contracts (60.0% and 65.8% for women; p<0.001 and 68.9% and 68.8% for men; p<0.001). Full-time contracts were also associated with higher satisfaction, with 75.0% of satisfied women and 89.5% of satisfied men holding full-time contracts (p=0.009 and p<0.001, respectively).

Table 3 presents the results of a multivariate weighted linear regression analysis, with job dissatisfaction as the dependent variable and survey year as the independent variable, adjusted for sociodemographic and work-related variables, and stratified by sex.

**Table 3:** Adjusted multivariable weighted linear regression analysis of job dissatisfaction change (2016 vs 2022) by sex *Statistical significance: p-value < 0.05

	Women			Men		
	β	95% CI	p-value*	β	95% CI	p-value*
Year (ref. 2016)	-0.02	-0.03;0.00	0.012	-0.02	-0.03;0.00	0.008
Age	0.00	0.00; 0.00	0.377	0.00	0.00; 0.01	0.082
Born outside Spain	0.09	0.02; 0.16	0.012	0.02	-0.05; 0.08	0.595
Social class (ref. manual)	-0.09	-0.16; -0.03	0.005	-0.10	-0.16; -0.04	0.002
Full-time contract	-0.03	-0.10;0.04	0.452	-0.11	-0.21; -0.02	0.022
Job status (ref. lifelong contract)						
Temporary contract	0.10	0.01;0.19	0.024	0.09	0.00;0.18	0.057
No contract	0.19	0.01;0.37	0.042	0.36	0.15; 0.57	0.001
Self-employment	0.06	-0.05;0.17	0.271	-0.03	-0.11; 0.06	0.577
Company owner	-0.29	-0.48; -0.09	0.004	-0.21	-0.35; -0.08	0.002

Overall, job dissatisfaction decreased significantly for both women and men. Non-manual employment was associated with reduced dissatisfaction (women: -0.09; 95% CI -0.16 to -0.03; men: -0.10; 95% CI -0.16 to -0.04). Women born outside Spain reported increased dissatisfaction (0.09; 95% CI 0.02 to 0.16).

**Figure 1:**
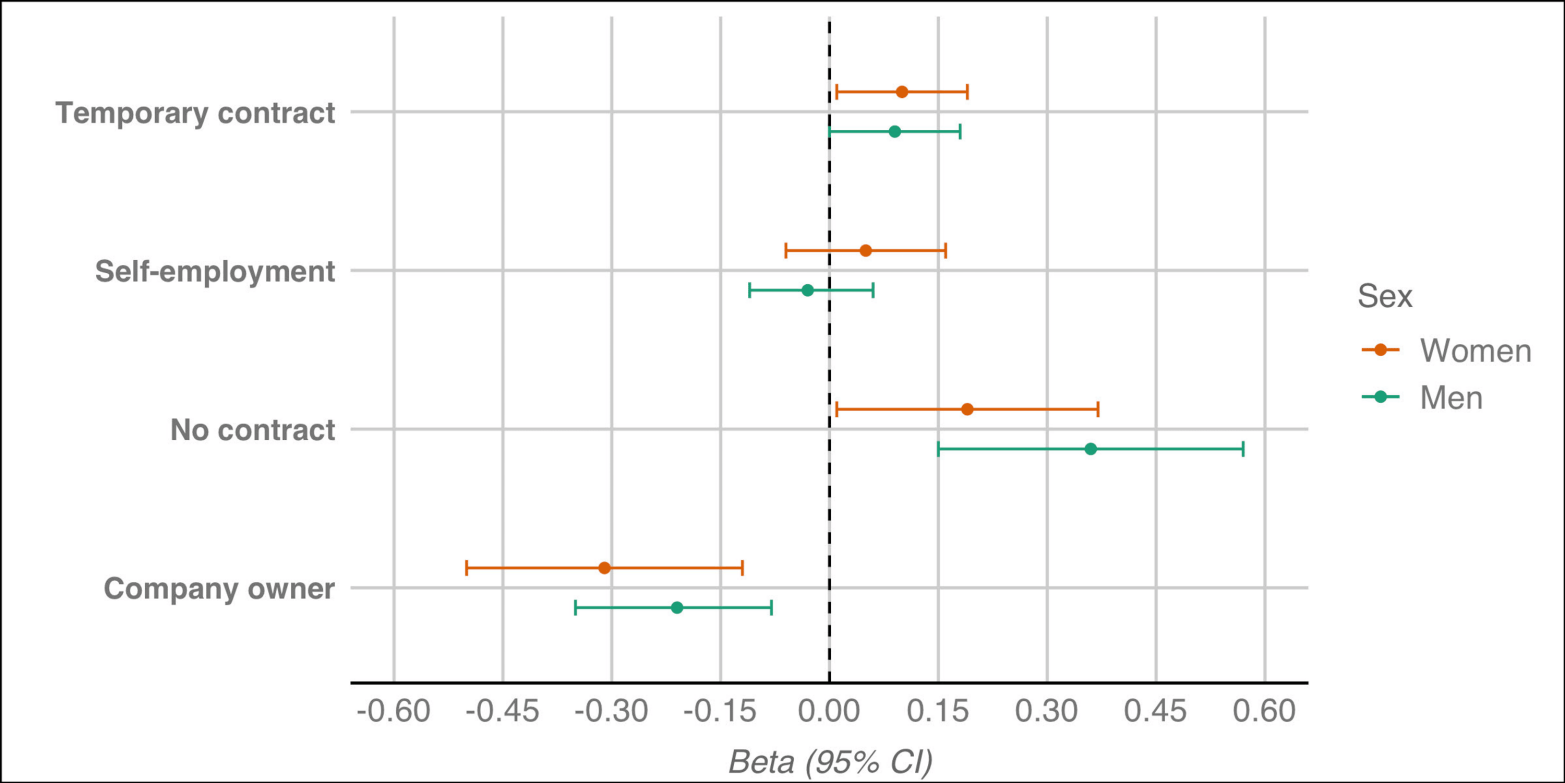
Association of different types of job status with changes in job dissatisfaction between 2016 and 2022

When it comes to work-related variables, full-time contracts were associated with reduced dissatisfaction, significant only for men (-0.11; 95% CI -0.21 to -0.02). Women with temporary contracts reported increased dissatisfaction (0.10; 95% CI 0.01 to 0.19). For both women and men, having no contract was associated significantly to an increase in job dissatisfaction, while being a company owner implied an increase. The association of different types of job status with changes in job dissatisfaction is depicted visually in Figure 1. 

**Figure 2:**
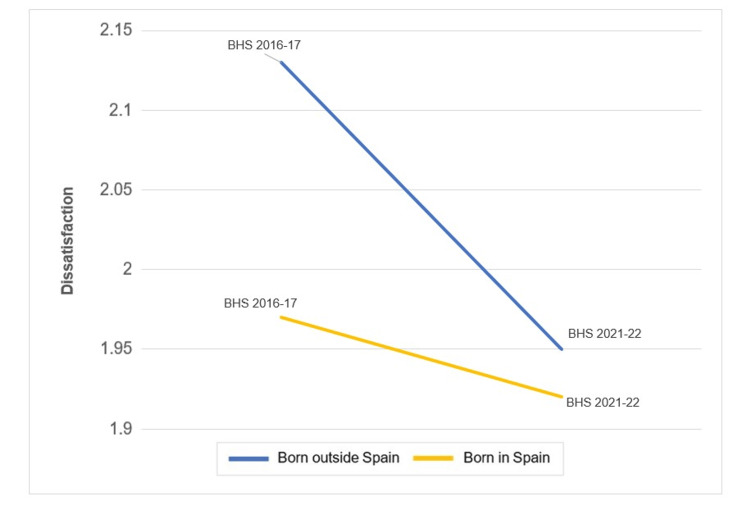
Comparison between mean dissatisfaction level in individuals born in Spain and outside Spain (p for interaction=0.032)

The increased trend observed in dissatisfaction levels between the surveys conducted in 2016 and 2022 was modified by birthplace (p for interaction=0.032) (Figure 2). Notably, the stratified analysis by the levels of the modifier showed a higher decrease in work dissatisfaction among individuals born outside Spain (-0.03; 95% CI -0.05 to -0.01) compared to native-born individuals (-0.01; 95% CI -0.02 to 0.00). This analysis is shown in Table 4.

**Table 4:** Adjusted multivariable weighted linear regression analysis of job dissatisfaction change (2016 vs 2022) by place of birth *Statistical significance: p-value < 0.05

	Born outside Spain			Born in Spain		
	β	95% CI	p-value*	β	95% CI	p-value*
Year (ref. 2016)	-0.03	-0.04; -0.01	0.001	-0.01	-0.02;0.00	0.085
Age	0,00	0.00; 0.00	0.615	0,00	0.00; 0.00	0.197
Sex (ref. women)	-0.05	-0.13;0.03	0.201	-0.02	-0.07; 0.04	0.539
Social class (ref. manual)	0.13	0.05;0.21	0.001	0.10	0.03;0.16	0.002
Full-time contract	0.16	0.06;0.25	0.001	0,00	-0.07;0.07	0.997
Job status (ref. lifelong contract)						
Temporary contract	0.08	-0.02;0.18	0.099	0.10	0.02;0.18	0.016
No contract	0.28	0.12;0.44	0.001	0.09	-0.17;0.34	0.512
Self-employment	0.03	-0.10;0.15	0.689	0.01	-0.07;0.10	0.758
Company owner	-0.18	-0.40;0.04	0.112	-0.24	-0.37; -0.11	<0.001

## Discussion

The presented results show an improvement in job status and a general decrease in job dissatisfaction after the pandemic. However, these results are not distributed homogeneously, as differences have been identified based on sex, social class and working conditions. Overall, this study supports that the labour market in Barcelona has experienced changes after the COVID-19 pandemic, which aligns with other published academic work related to similar settings [25,26].

The higher proportion of individuals with non-manual jobs [across women and men] and men born outside Spain could have influenced the results of our study. People with non-manual jobs, which usually require a higher level of education, are more likely to have better contractual situations and higher job satisfaction [27,28], while migrants are generally linked to poorer working conditions [29]. This change in the number of individuals born outside Spain could be due to the general migratory trend in Barcelona, which saw an increase in migrated population between 2017 and 2021 [30].

In our study, we also observed that men referred lower financial strain in the 2021-2022 BHS which might be because pay increases tend to benefit men more than women [31]. It is also important to point out that the minimum wage increased steadily in Spain between 2018 and 2022, potentially leading to a positive effect on people’s perceived financial strain. Employment conditions in Barcelona also show a generalised improvement, with an increase in lifelong contracts. This change is only significant among men, who also saw a decrease in their hours of paid labour per week. This aligns with existing research showing that women tend to benefit less from workplace changes, highlighting a gender [31]. Some countries saw a decrease in the quality of labour-related indicators after the COVID-19 pandemic [25], but in some cases, others displayed the protective effect work-related public policies like those applied in Spain, and subsequently in Barcelona [32].

The analysis also shows that a higher burden of domestic labour seems to be placed on women, which is well-documented in the literature and consistent with widespread gender disparities [6,33]. Our research found that men are now spending more time on household chores. This could be related to the rise in remote work during the pandemic, which has been identified as a potential factor that could increase men's involvement in domestic tasks [34] or a self-serving bias affecting men’s reported answers.

A change in job dissatisfaction

Regarding job dissatisfaction, individuals living in Barcelona have experienced a decrease after the pandemic. This is consistent with other evidence suggesting that individuals were less satisfied before the pandemic, with a change occurring during the period 2020-21, when working from home became more predominant [35]. It is important to note that job satisfaction is complex and demonstrates associations with diverse aspects of a person’s life [27,36]. Likewise, the increase found in our study is influenced by sex, and other sociodemographic characteristics, and work-related variables.

Despite women generally benefitting less than men from the observed work-related improvements, our study found they still experienced a decrease in job dissatisfaction. This could be due to the well-documented phenomenon, known as the “gender job-satisfaction paradox”. This paradox refers to women experiencing higher levels of satisfaction at their workplace than men despite generally lower wages, fewer opportunities for professional advancement, and fewer chances of promotion [37,38]. However, women in the labour market are not a homogeneous group, and this paradox does not apply to all women. The role they perform at their workplace as well as their education level can alter the effect of the paradox [39,40].

In Barcelona, this might apply to women born outside Spain, as they referred to an increase in job dissatisfaction. This indicates that the adverse work-related conditions that migrants experience can determine their job satisfaction [29,41]. Social class has also been shown to be a determinant of job satisfaction [2,19,35]. Barcelona seems to be no exception, as our results reflected an increase in job dissatisfaction among both women and men engaged in manual labour, which contrasts with the general decrease among all individuals.

Current literature does not clearly specify the role the pandemic has had in the deterioration of job satisfaction among manual workers. The results obtained in terms of job dissatisfaction could reflect the direct effects of COVID-19 on workers more affected by social inequalities [13], with the mid-term and long-term impacts remaining uncertain. 

The improvements in the labour market of Barcelona could have influenced the observed decrease in job dissatisfaction. While our results show a higher job satisfaction linked to full-time contracts, some evidence supports that working part-time can indeed reinforce job satisfaction due to higher flexibility and less exposure to injury risk [42]. However, the available research is generally inconclusive on whether working full-time has a clear impact on job satisfaction, indicating that it can be influenced by other work-related conditions, such as salary, job security, or workplace organisation [43].

Additionally, despite mixed evidence, the lower job satisfaction shown among women with temporary contracts and individuals with no contract in Barcelona aligns with other research. A study conducted in Germany concluded that low job security is detrimental to workers’ well-being, despite showing some benefits in the form of a “honeymoon phase” [44].

Generally, there have been positive shifts in the post-pandemic labour market of Barcelona and a decrease in job dissatisfaction among its citizens. The multiple labour policies and reforms approved at the national level in Spain since 2018 could have helped prevent a negative impact of COVID-19 on the labour market.

Implications for public health policy

The findings of this study underline relevant public health policy implications, particularly concerning job dissatisfaction. Job dissatisfaction is a recognised social determinant of health, closely associated with adverse mental health and general well-being outcomes [10]. Our results demonstrate that, despite a general improvement in job satisfaction in Barcelona following the pandemic, significant disparities persist. Women born outside Spain, individuals engaged in manual labour, and those in precarious employment conditions reported higher levels of job dissatisfaction compared to the general population. These groups are likely to experience chronic stress due to job insecurity, poor working conditions, and limited access to resources, which may exacerbate existing health inequities [45]

These findings highlight the need for targeted public health policies and interventions to mitigate the impact of unfavourable working conditions. Policies should prioritize reducing job precariousness, promoting gender equity in the labour market, and supporting migrant workers through tailored programmes, addressing broader determinants such as the balance between paid and domestic labour remains essential, particularly for women, who continue to bear the brunt of unpaid caregiving responsibilities [19].

Integrating social determinants of health into labour market policies and promoting intersectoral collaboration can play a pivotal role in reducing inequalities, improving worker satisfaction, and enhancing overall population health [3].

Limitations

Our study has some limitations. One limitation is related to the available variables. While the 2016-17 and 2021-22 BHS are generally similar, some questions have changed, limiting the number of comparable sociodemographic and work-related variables. This is particularly relevant for the variant “gender identity” which is only present in the 2021-22 BHS and therefore could not be used to stratify; instead, we used “assigned sex at birth”. Moreover, self-reported answers regarding lack of financial strain and domestic labour distribution could be influenced by self-serving bias. In addition, while the 4-point scale used for job dissatisfaction can be treated as continuous given the large sample size, this approach could introduce potential bias.

It is important to note though that the samples used for both surveys were selected following the same well-researched methodology, making them comparable and contributing to the validity of our study [24]. As a social determinant of health, sex was consistently used for stratification, allowing us to present results in a more nuanced manner. Any interpretation related to sociodemographic and work-related variables needs to consider the potential differences between women and men [46].

Additionally, few studies have addressed the changes in the labour market after the pandemic, and evidence on job satisfaction determinants is contradictory, making it difficult to reach sound conclusions supported by previous research. However, the limited evidence makes our findings particularly relevant. As work-related conditions are essential in determining the well-being of individuals and are recognised as SDH [2], the provided evidence helps understand how these conditions have shifted and how these changes could potentially widen inequalities post-COVID-19.

Our study has used a representative weighted sample and a solid analysis to provide relevant evidence, particularly regarding changes in the labour market and job dissatisfaction. This study sets the ground for further improved research with the upcoming BHS. 

## Conclusions

The labour market in the city of Barcelona has experienced changes between 2016 and 2022. These shifts can be partially attributed to various labour policies implemented from 2018 to 2022. Additionally, the negative impact of the COVID-19 pandemic may have further influenced these changes. There has been a general improvement in the job status of citizens and a better distribution of domestic labour between women and men. However, these improvements are not distributed homogeneously across the population. Women have benefited less from them, thus maintaining the existing gender gap in the labour market. As per job dissatisfaction, despite a generalised decrease in line with a lower financial strain, migrant women, individuals in manual jobs, and those with more precarious contractual situations remain less satisfied with their post-pandemic job conditions.

Overall, the general positive effects dilute once observed through different axes of inequality. Any measures implemented to protect workers and the labour market, both in response to COVID-19 and beyond, must incorporate a perspective based on social determinants of health, with a particular emphasis on addressing existing gender inequalities.
